# Supplementary material for: Lactose Residual Content in PDO Cheeses: Novel Inclusions for Consumers with Lactose Intolerance
Source: Foods. 2021 Sep 21;10(9):2236. doi: 10.3390/foods10092236 (PMC8464992; doi:10.3390/foods10092236)
Supplement: Supplementary file 1 [file foods-10-02236-s001.zip › foods-1313812 sup/Supplemental materials_Table S1 (a)-1.pdf]

Questionnaire administrated to lactose intolernat consumers

Question	Answers
Are you lactose intolerant?	Yes
	I am a parent or guardian of a lactose intolerant minor
	No
How did you find out you are lactose intolerant?	Diagnosed by breath test and/or specific genetic test
	Auto-diagnosis
	Not-validated tests (e.g. Vega-Test, Cito-Test)
How long have you known you are lactose intolerant?	Since birth
	Less than 1 year
	1-3 years
	More than 3 years
What is your gender?	Male
	Female
What is your age?	Less than 18 years
	18-24 years
	25-34 years
	35-44 years
	45-54 years
	More than 55 years
What is the Italian region you live in?	Abruzzo
	Basilicata
	Calabria
	Campania
	Emilia Romagna
	Friuli-Venezia Giulia
	Lazio
	Liguria
	Lombardia
	Marche
	Molise
	Piemonte
	Puglia
	Sardegna
	Sicilia
	Toscana
	Trentino-Alto Adige
	Umbria
	Valle d'Aosta
	Veneto

What is your occupation?	Employee
	Student
	Non-resident student
	Stay-at-home
	Retired
Do you consume naturally lactose-free cheeses and/or "delactosed" cheeses?	Yes, both
	Yes, only naturally lactose-free cheeses
	Yes, only "delactosed" cheeses
	Rarely
	Never
Which are the PDO cheeses that you buy the most?<br>Choose a maximun of three options.	Grana Padano and Parmigiano Reggiano long-aged (30-36 months)
	Grana Padano and Parmigiano Reggiano (any ageing)
	Gorgonzola
	Emmentaler
	Pecorino
	Fontina
What is your knowledge about the difference between naturally lactose-free cheeses and "delactosed" cheeses?	Scarce
	Sufficient
	Good
	Excellent
How often do you understand if PDO cheese is naturally lactose-free from its label?	Rarely
	Sometimes
	Always
Do you think a list of naturally lactose-free cheeses would be useful to consult?	Not at all
	Yes, but I wouldn't use it
	Yes, it could be useful
	Yes, it is necessary
