# Supplementary material for: Lactose Residual Content in PDO Cheeses: Novel Inclusions for Consumers with Lactose Intolerance
Source: Foods. 2021 Sep 21;10(9):2236. doi: 10.3390/foods10092236 (PMC8464992; doi:10.3390/foods10092236)
Supplement: Supplementary file 1 [file foods-10-02236-s001.zip › foods-1313812 sup/Supplemental materials_Table S1 (b).pdf]

Questionnaire administrared to professionals of nutrition	
**Question**	**Answers**
What is your occupation?	Nutritionist
	Dietician
	Medical doctor specialized in nutrition
How long have you been exercising your professional activity?	0-3 years
	4-10 years
	More than 10 years
Which are the Italian regions you work in?	Abruzzo
	Basilicata
	Calabria
	Campania
	Emilia Romagna
	Friuli-Venezia Giulia
	Lazio
	Liguria
	Lombardia
	Marche
	Molise
	Piemonte
	Puglia
	Sardegna
	Sicilia
	Toscana
	Trentino-Alto Adige
	Umbria
	Valle d'Aosta
	Veneto
How many are your patients in total? Indicate the numer in the year 2019.	Less than 50
	51-100
	101-200
	More than 200
How many are your lactose-intolerant patients? Indicate the numer in the year 2019.	Less than 25
	26-50
	51-100
	More than 100
What is your knoledge about lactose intolerance topic?	Scarce
	Sufficient
	Good
	Excellent, acquired thaks to certified training courses

Which test did your lactose intolerant patients are mailny diagnosed by when you first visited them?	H2 Lactose Breath test
	LCT C/T-13910 Gentic test
	Both above-mentioned tests
	Auto-diagnosis
	Not-validated tests (e.g. Vega-Test, Cito-Test)
How much clarity do you think there is about the topic "lactose intolerance and naturally lactose-free cheeses"?	Scarce
	Sufficient
	Good
	Excellent
Which are the naturally lactose-free PDO cheese that you advise to your lactose intolerant patients the most? Choose a maximum of three options.	Grana Padano and Parmigiano Reggiano long-aged (30-36 months)
	Grana padano and Parmigiano Reggiano (any ageing)
	Gorgonzola
	Emmentaler
	Pecorino
	Fontina
Do you think the nowdays labeling policy for naturally lactose-free PDO cheeses to be satisfying?	Scarce
	Sufficient
	Good
Do you think a table of naturally lactose-free cheeses would be useful to distribute to your lactose intolerant patients?	Not at all
	Yes, but I wouldn't use it
	Yes, it could be useful
	Yes, it is necessary
Do you know about the Italian lactose-intolerants patients' association, AILI (Associazione Italiana Latto-Intolleranti)?	No
	I have heard of it
	Yes
How did you learn about the Association?	Personal research on Google
	Event (stands, trade fairs, conventionals)
	Social media (Facebook, Instagram)
	Word-of-mouth
